# An Erythritol-Sweetened Beverage Induces Satiety and Suppresses Ghrelin Compared to Aspartame in Healthy Non-Obese Subjects: A Pilot Study

**DOI:** 10.7759/cureus.11409

**Published:** 2020-11-10

**Authors:** Zachary A Sorrentino, Garrett Smith, Lindsey Palm, Kartik Motwani, John Butterfield, Christian Archer, Rebecca Henderson, Coy Heldermon, Shiva Gautam, Mark L Brantly

**Affiliations:** 1 Medicine, University of Florida College of Medicine, Gainesville, USA; 2 Medicine, University of Florida College of Medicine, Jacksonville, USA

**Keywords:** erythritol, aspartame, ghrelin, osmolarity, appetite and sateity, artificial sweetener, treating obesity

## Abstract

Despite the reduced caloric content of artificially sweetened beverages (ASBs) relative to those sweetened with sucrose, consumption of ASBs fail to consistently decrease the risk of obesity and associated diseases. This failure may be due to the inability of ASBs to effectively reduce appetite and hence overall caloric intake. A variety of non-nutritive sweeteners (NNS), however, remain to be screened for effectiveness in promoting satiety and reducing calorie consumption. Erythritol is well-tolerated, low-calorie sugar alcohol widely used as a sugar substitute. It is unique among NNS due to its low sweetness index relative to glucose, meaning that it is typically served at much higher concentrations than other common NNS. Animal and human studies have noted correlations between osmolarity, satiety, and levels of satiety hormones, independent of the effects of sweetness or nutritive value. We hypothesized that consumption of a beverage sweetened with erythritol to the sweetness and osmolarity of a common soft drink will improve self-reported satiety and more strongly affect the magnitude of changes in the hormone ghrelin than would an iso sweet beverage sweetened only with aspartame, a sweetener with a high sweetness index relative to glucose. Using a randomized double-blind crossover trial, we found that serum ghrelin was significantly decreased after consumption of an erythritol-sweetened beverage compared to aspartame. Likewise, consumption of the erythritol-sweetened beverage increased various measures of satiety in volunteers. Knowledge gained from this project demonstrates that high-osmolarity NNS may be useful in formulating ASBs that are satiating and low in calories.

## Introduction

Low-calorie artificially sweetened beverages (ASBs) have been used in place of sucrose-sweetened drinks for decades, but their use is not typically associated with lower obesity rates, improved satiety, or modulation of physiologic satiety markers [1-3]. ASBs also have not been shown to decrease the incidence of type 2 diabetes compared to sugar-sweetened beverages, and may even be linked to exacerbation of cardiometabolic risk factors [4]. The use of non-nutritive sweeteners (NNS) is increasing, particularly in the sweetening of beverages, and many users have the goal of decreasing caloric intake and/or achieving weight loss [5]. As most studies indicate that widely used NNS is not highly effective in decreasing calorie intake, further research is needed to identify effective NNS based on physiologic determinants of satiety.

Mechanisms underlying satiety are complex, and sweetness alone is not sufficient to trigger satiety associated hormonal changes, as ASBs similar in sweetness to caloric beverages fail to appreciably suppress ghrelin or induce the release of other satiety-associated hormones such as glucagon-like peptide-1 (GLP-1), gastric inhibitory peptide (GIP), and peptide tyrosine tyrosine (PYY) [1,6]. In contrast, glucose induces the release of satiety hormones and suppression of ghrelin, congruent with research indicating that satiety hormone modulation depends on many other factors besides taste [1,6]. Beyond satiety hormones, consumption of ASBs also activates different neural reward pathways than the consumption of sucrose-sweetened beverages [7]. Considering these findings, additional properties of NNS besides sweetness should be considered in predicting efficacy in satiety induction.

Erythritol is a natural, low-calorie (0.2 kilocalories/gram (Kcal/g)) sugar alcohol that may promote satiety more effectively than other NNS [8]. Erythritol can be rapidly absorbed by the gastrointestinal (GI) tract in quantities of up to 50 g in the average adult per day with little to no side effects and is readily excreted by the kidneys with minimal metabolism; children also tolerate erythritol well [8-11]. Additionally, consumption of erythritol has been found to decrease the incidence of dental caries in children and improve markers of vascular function in diabetic patients [12,13]. Unlike other NNS, erythritol has a sweetness index close to that of sucrose (sweetness index of approximately 70% relative to sucrose [10]). Many other NNS such as aspartame and sucralose have sweetness indices more than 100 times that of sucrose and consequently are used at very low osmolarities [10]. In contrast, erythritol must be used at a much higher osmolarity than other NNS to achieve iso sweetness to sucrose, which may better enable it to promote satiety at common doses. Osmolarity has been shown to induce alterations in satiety-related hormones consistent with reduced hunger independently of caloric content. In animal and human subjects, injection of non-caloric, high-osmolarity solutions into the duodenum had effects comparable to glucose in suppressing serum ghrelin and increasing GIP, GLP-1, and PYY [14-16]. Similarly, intragastric infusion of hyperosmolar solutions in various models resulted in slowed gastric emptying and reduction of subsequent food intake, which is also satiety markers [17-20].

Due to strong evidence from human and animal studies that increased osmolarity in the duodenum could contribute to functional and hormonal markers of satiety, we hypothesized that a beverage sweetened with erythritol would be superior in inducing satiety as compared to ASBs sweetened with the commonly used NNS aspartame. In a randomized double-blind crossover trial of human subjects, we compared the effects of a high osmolarity erythritol-sweetened solution to a low osmolarity aspartame-sweetened solution at equal sweetness and volume on serum ghrelin and subjective hunger.

## Materials and methods

Participants

All participants provided written and ongoing informed consent over the course of the trial. The targeted study population was adults aged 18-40 years, who were non-overweight (body mass index (BMI) of 18.5-24.9) and without pre-existing medical conditions and with stable body weight for three months prior to enrollment. Additional exclusion criteria included the presence of dietary restrictions, prescription medication usage besides oral contraceptives, supplement usage besides multivitamins, pregnancy (urine human chorionic growth hormone (hCG) test required at all study visits), and evidence of abnormal medical conditions upon physician-conducted medical history and physical examination. Participants were recruited via flyers posted in public areas on the University of Florida (UF) campus. The study was conducted in the outpatient research facility at the UF Clinical and Translational Science Institute (CTSI), under medical supervision. Statisticians at the UF Department of Biostatistics, Epidemiology, and Research Design (BERD) concluded that 12 participants would need to complete the trial to detect a significant difference in the primary endpoint (total serum ghrelin suppression) at p=0.025 and 80% power. The demographic and physical examination information is summarized (Table 1) for participants who completed the trial. 

**Table 1:** Baseline characteristics of study participants Results are n (%) or mean ± SD.

Demographics	
Variable	Number
Participants completed	12
Sex (male)	5 (42%)
Race (non-white)	5 (42%)
Age (years)	25.4 ± 2.4
Body mass index	21.7 ± 1.9
Systolic blood pressure (mmHg)	110.9 ± 8.4
Diastolic blood pressure (mmHg)	70.9 ± 7.0

Study design

**Figure 1:**
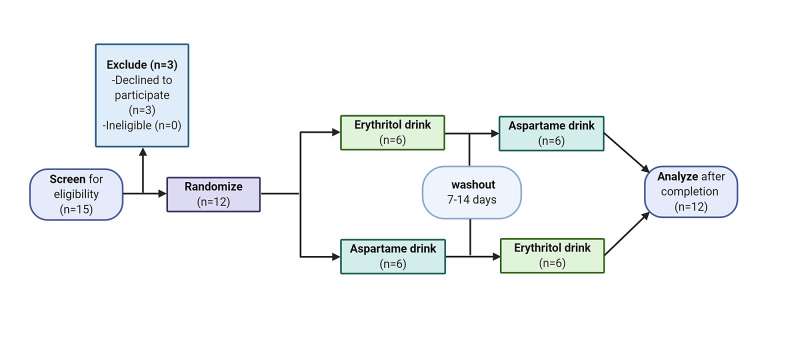
Consort diagram describing the trial design and enrollment Twelve participants were enrolled in the study and randomized to each beverage sequence. None of the 12 participants withdrew before study completion.

The study utilized a double-blinded, randomized two-way crossover design. Participants first underwent a screening visit at which informed consent was obtained and exclusion criteria were assessed. Participants were then randomized to one of two arms; randomization and allocation were performed by random length permuted blocks by a BERD statistician (Figure 1). On each of two separate experimental visits, participants received oral doses of erythritol or aspartame-sweetened beverages. The two treatments were separated by a 7-14-day washout period for each participant. Study investigators and participants were blinded as to which drink was consumed until after all data analysis was performed following completion of the trial by all participants. The study design is summarized in a consort diagram (Figure 1). 

Preparation of solutions

Participants received oral doses of one of two dietary grade sweeteners fully dissolved in 250 mL of room temperature tap water: (1) aspartame or (2) erythritol. The concentrations used for aspartame (185 mg, 2.5 mM) and erythritol (50.8 g, 1.66 M) were based on the osmolarity and sweetness of 75 g of glucose in 250 mL aqueous solution, which is the standard for an oral glucose tolerance test. Beverages were adjusted to be iso sweet with similarly low caloric content (<10 calories); the two solutions were approximately 70% the sweetness of a comparable sucrose solution [8]. Solutions were prepared by CTSI staff 30 minutes prior to consumption at each participant's session and served in vessels concealing the appearance of the liquid.

Study procedure

Subjects were instructed to fast overnight (>10 hours) and abstain from caffeine and strenuous exercise prior to each experimental visit. Upon arrival, participants were fitted with an intravenous cannula in an antecubital vein from which all blood samples would be collected. At all times, one registered nurse per participant was present to conduct blood draws and monitor patients. Following the collection of the baseline blood sample and hunger ratings, participants orally consumed the test solution within two minutes. Approximately 2 mL of blood was collected at baseline and at 10, 15, 20, 30, 45, 60, and 90 minutes post beverage consumption. Times were chosen based on peak ghrelin suppression postprandially [1]. Participants’ hunger levels were assessed using a linear scale [21] administered by study investigators at times of 0 (baseline), 30, and 120 minutes post beverage consumption. Subjects had no exposure to food cues during or before blood collection. Participants were awake and relaxed in either a seated or supine position for the duration of each experimental visit. Vital signs were monitored at the beginning and end of each testing session. 

Determination of total serum ghrelin ELISA 

The serum was isolated from collected blood for subsequent ghrelin enzyme-linked immunosorbent assay (ELISA) analysis. To prepare the serum, whole blood was drawn directly from the intravenous cannula into a pre-chilled Vacutainer® tube (BD, Franklin Lakes, NJ, USA) containing EDTA (ethylenediaminetetraacetic acid). Samples were kept on ice at all times. Immediately after collection, 4-(2-aminoethyl)-benzenesulfonyl fluoride hydrochloride (AEBSF, Millipore Sigma, Temecula, CA, USA) was added to a final concentration of 1 mg/mL. Blood was promptly centrifuged at 2000 g for 15 minutes at 4°C, after which serum was isolated and stored at -80°C.

Sandwich ELISA kits specific for total human ghrelin (RAB1052, Sigma-Aldrich, St. Louis, MO, USA) were utilized to assay total ghrelin levels (both acylated and de-acylated forms) from collected serum samples according to the manufacturer’s protocol. All assays were performed in triplicate for each sample and over the same two-day period. Ghrelin concentration was determined by measuring absorbance at 450 nm (all absorbances were background subtracted); ghrelin standards in triplicate (0, 51.2, 128, 320, 900, and 2000 pg/mL) were used for the construction of a calibrated concentration curve (Graphpad Prism, San Diego, CA, USA) to determine total human ghrelin concentration for each sample.

Statistical analysis

The primary outcome (total serum ghrelin) was measured as the change from baseline values for each patient visit, then the area under the curve (AUC) was calculated for each patient visit using the trapezoidal rule from t=10 to t=90 minutes. AUC was compared between the beverage groups using a Wilcoxon signed-rank test given the small sample size and lack of normality. This approach, known as response feature methods, first summarizes repeated observations into a single measure and then compares these summaries using common statistical methods. Nadir of serum ghrelin concentration was also compared between treatment groups using a Wilcoxon signed-rank test. Since the response feature approach may not reflect changes in data over time, we also analyzed the primary outcome using a linear mixed model. Data were log-transformed before analysis in the mixed linear model.

Secondary outcomes for hunger were measured at 30- and 120-minute time points. Hunger scores were adjusted to the absolute percent change from baseline and compared by a linear mixed-effects model. Mean scores were calculated for each patient and beverage, and these summary data were used to compare beverage effects using the Wilcoxon signed-rank test. One missing data point (non-baseline) from the hunger surveys collected was substituted with the mean for that beverage group among participants of the same race. Given the exploratory nature of this study, corrections were not made for multiple comparisons. All analyses were performed using SAS/STAT software (SAS version 9.3, SAS Institute, Cary, NC, USA).

## Results

Total serum ghrelin

**Figure 2:**
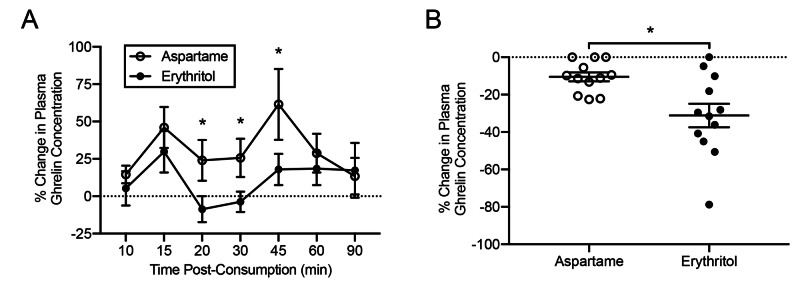
Changes in serum total ghrelin and hunger relative to baseline following the consumption of erythritol- or aspartame-sweetened beverages (A) Consumption of an erythritol beverage was associated with a significantly reduced serum ghrelin concentration compared to consumption of an aspartame beverage at the 20, 30, and 45 minutes time points. (B) Erythritol consumption was associated with a mean reduction in ghrelin nadir compared to aspartame. Dotted line indicates the baseline value. Error bars represent the standard error of the mean. *=p<0.05.

Following administration of test beverages, collection of serum samples at various time-points, and ELISA detection of total ghrelin, ghrelin suppression was compared between the aspartame and erythritol beverage groups. Utilizing the linear mixed-effects model, a significant reduction in serum ghrelin relative to baseline was observed following consumption of erythritol versus aspartame (14% reduction, 95% CI=19%, 7%, p=0.0001), with a statistically significant reduction in serum ghrelin seen at time points 20 (p=0.007), 30 (p = 0.02) and 45 minutes (p=0.02) (Figure 2A). Nadir of total ghrelin was significantly reduced in patients consuming erythritol relative to aspartame (p = 0.01, F = 9.4, fixed effects, Figure 2B). AUC of serum total ghrelin concentration was not significantly different between participants consuming beverages sweetened with aspartame or erythritol (p=0.15).

Hunger

**Figure 3:**
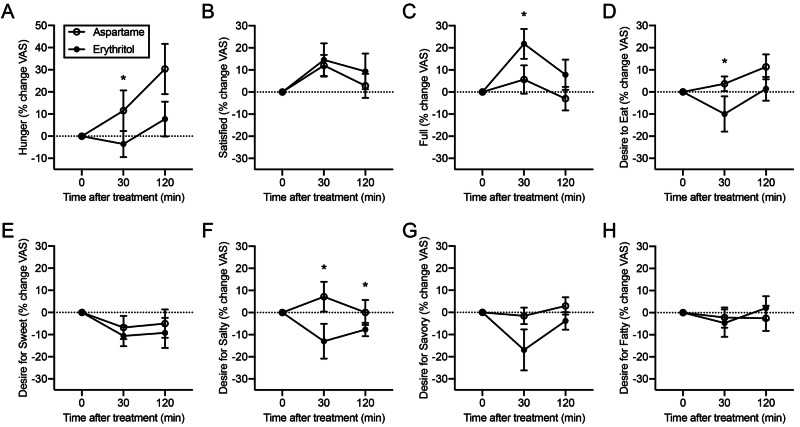
Changes in hunger ratings relative to baseline following consumption of erythritol or aspartame beverages Significant differences between the beverage groups are seen at the 30-minute time point for (A) hunger, (C) fullness, (D) desire to eat, and (F) desire for salty foods. No differences between the groups were seen for (B) satisfaction, (E) desire for sweet foods, (G) desire for savory foods, or (H) desire for fatty foods. Dotted lines indicate baseline values. Error bars represent the standard error of the mean. *=p<0.05.

Hunger ratings were assessed at three time-points throughout the trial using visual analogue scales. Ratings were compared between the erythritol and aspartame groups. Wilcoxon signed-rank tests of self-reported hunger indicated that participants receiving erythritol-sweetened beverages, compared to those consuming an aspartame-sweetened beverage, reported feeling less hungry (p=0.03), fuller (p=0.03), a reduced desire to eat (p=0.04), and a reduced desire for salty foods (p=0.03) at the 30-minute time point; and a reduced desire for salty foods (p=0.04) at the 120-minute time point (Figure 3). No change was identified for other measures of hunger between the beverage groups or at other time points. There was no significant interaction of beverage and day of visit for any hunger measure and results were unaffected by visit order.

## Discussion

Ghrelin is a 28 amino acid peptide hormone released by the GI tract that stimulates short-term hunger. Although its regulatory mechanisms are incompletely understood, circulating total ghrelin is generally related to postprandial satiety, as ghrelin is suppressed following a meal [6,22-25]. The degree of ghrelin suppression generally increases with caloric content and satiety, although other factors are likely important [6,26-28]. Previous studies have observed that increasing osmolarity in the duodenum independently suppresses ghrelin and induces the release of other satiety hormones [14-16]. Thus, in this study, the high osmolarity of the erythritol beverage is likely the mechanism behind its significant ghrelin nadir suppression compared to the aspartame beverage and significantly increased satiety measures after ingestion of the erythritol beverage compared with the aspartame beverage likely follow from the increased ghrelin suppression. Variability in ghrelin response both over time and at the nadir may suggest that different hormonal responses to erythritol exist between individuals, however even with variability in individuals and in ghrelin measurements then the ghrelin levels for erythritol were consistently below that of aspartame at all time points, although this did not reach significance at some time points. Our results suggest that usage of NNS in ASBs that are hundreds of times sweeter than sucrose may not be effective in inducing satiety due to the low osmolarities at which they are used, and usage of high osmolarity NNS such as erythritol in ASBs should be considered [10].

Though the effect of osmolarity on satiety in humans has yet to be firmly established, this information may help in the formulation of ASBs designed to help curb overall caloric consumption. The low-calorie sweetener erythritol appears to be particularly promising in that it can be tolerated in high concentrations while having a similar sweetness index to sucrose [8,9,11]. Two other trials have studied the linkage between erythritol and markers of satiety in different contexts [29]. One study found that partial replacement of sucrose with erythritol in a solid meal led to participants consuming fewer calories compared with the sucrose only breakfast [29]. Likewise, intra-gastric infusion of erythritol resulted in significantly increased GLP-1 and CCK release along with slowed gastric emptying in volunteers, which further indicates the satiating effect of erythritol [30].

## Conclusions

In summary, this pilot study for the first time demonstrates that usage of the high-osmolarity erythritol is efficacious in inducing satiety and suppressing ghrelin when consumed orally in a non-caloric beverage; this finding is of value in formulation of beverages that are simultaneously satiating and low in calories. 
